# Agonists for G-protein-coupled receptor 84 (GPR84) alter cellular morphology and motility but do not induce pro-inflammatory responses in microglia

**DOI:** 10.1186/s12974-017-0970-y

**Published:** 2017-10-03

**Authors:** Li Wei, Kyohei Tokizane, Hiroyuki Konishi, Hua-Rong Yu, Hiroshi Kiyama

**Affiliations:** 1 Department of Functional Anatomy and Neuroscience, Nagoya University, Graduate School of Medicine, Nagoya, 65 Tsurumai-cho, Showa-ku, Nagoya, Aichi 466-8550 Japan; 2 National Key Laboratory of Birth Defects and Reproductive Health, Chongqing Institute of Population and Family Planning, Chongqing, 400020 China; 3 College of Basic Medical Science, Chongqing Medical University, Chongqing, 400016 China

**Keywords:** G-protein-coupled receptor 84, Pro-inflammatory response, Microglia morphology, Microglia motility, Fatty acid

## Abstract

**Background:**

Several G-protein-coupled receptors (GPCRs) have been shown to be important signaling mediators between neurons and glia. In our previous screening for identification of nerve injury-associated GPCRs, G-protein-coupled receptor 84 (GPR84) mRNA showed the highest up-regulation by microglia after nerve injury. GPR84 is a pro-inflammatory receptor of macrophages in a neuropathic pain mouse model, yet its function in resident microglia in the central nervous system is poorly understood.

**Methods:**

We used endogenous, natural, and surrogate agonists for GPR84 (capric acid, embelin, and 6-OAU, respectively) and examined their effect on mouse primary cultured microglia in vitro.

**Results:**

6-n-Octylaminouracil (6-OAU), embelin, and capric acid rapidly induced membrane ruffling and motility in cultured microglia obtained from C57BL/6 mice, although these agonists failed to promote microglial pro-inflammatory cytokine expression. Concomitantly, 6-OAU suppressed forskolin-induced increase of cAMP in cultured microglia. Pertussis toxin, an inhibitor of Gi-coupled signaling, completely suppressed 6-OAU-induced microglial membrane ruffling and motility. In contrast, no 6-OAU-induced microglial membrane ruffling and motility was observed in microglia from DBA/2 mice, a mouse strain that does not express functional GPR84 protein due to endogenous nonsense mutation of the *GPR84* gene.

**Conclusions:**

GPR84 mediated signaling causes microglial motility and membrane ruffling but does not promote pro-inflammatory responses. As GPR84 is a known receptor for medium-chain fatty acids, those released from damaged brain cells may be involved in the enhancement of microglial motility through GPR84 after neuronal injury.

## Background

G-protein-coupled receptors (GPCRs) form the largest superfamily of membrane proteins, and several GPCRs are important signal mediators between neurons and glia, protecting neurons from pathological stressors that cause disease [1, 2]. To identify GPCRs implicated in neuronal survival and nerve regeneration after neuronal injury, we previously performed a gene screen targeting GPCRs [3]. For this gene screen, we designed 274 primer sets for putative nonsensory GPCRs (including orphan receptors but excluding sensory GPCRs) and successfully identified several candidate GPCRs [3]. Among 274 genes, 29 genes were up-regulated by more than twofold after nerve injury. Further in situ hybridization secondary screening revealed that some of these receptors were specifically expressed and induced by microglia, and not neurons or astrocytes, in response to neuronal injury. This suggests that GPCRs and their ligands can be mediators for neuron-microglia interactions, particularly under traumatic or inflammatory conditions. Indeed, previous studies have demonstrated that C-C chemokine receptor type 5 (CCR5), CX3C chemokine receptor 1 (CX3CR1), and several purinergic receptors are expressed by microglia and play a variety of roles under physiological and pathological conditions [3–6].

In our previous GPCR screening study, mRNA encoding G-protein-coupled receptor 84 (GPR84) showed highest up-regulation (increased by approximately 47-fold) in microglia after neuronal injury [3]. Other papers also reported GPR84 up-regulation in microglia in a mouse model of endotoxemia, experimental autoimmune encephalomyelitis, and Alzheimer’s disease [7, 8]. GPR84 was first identified in myeloid cells by Wittenberger et al. [9], and separately by Yousefi et al. [10], and shown to be commonly expressed in species from lower vertebrates to humans. Interestingly a 2-bp frame-shift deletion in the second exon of the *GPR84* gene has also been discovered in some classical inbred mouse strains. This deletion generates a premature stop codon, resulting in a truncated protein that lacks transmembrane domains 4–7 [11]. Fourteen mice strains including DBA, NOD, and SJL are homozygous for the deletion, but other strains including C57BL/6 are intact. A subsequent knockout strategy using C57BL/6 mice demonstrated no obvious abnormalities except for certain immune responses [12]. However, the involvement of GPR84 in experimental neuropathic pain was recently reported [13]. This study revealed that pro-inflammatory responses of macrophages recruited to injured nerves are suppressed in *GPR84* KO mice after peripheral nerve injury, resulting in improvement of pain behavior. Subsequently, the authors concluded that GPR84 does not modulate microglial responses in the spinal dorsal horn, because GPR84 deficiency caused no alterations in microglial number and activation determined by ionized calcium-binding adapter molecule 1 and p-p38 immunostaining, respectively. Nonetheless, microglia responded to nerve injury with prominent up-regulation of GPR84 mRNA in the neuropathic pain model [13], as well as our motor nerve injury model [3], in which circulating macrophages do not contribute to the microglial pool [14–16], and thereby conflicts with the conclusion that there is no functional consequence of GPR84 deficiency in microglia [13].

Previous studies have deorphanized and characterized GPR84 as a receptor for medium-chain fatty acids such as capric acids [17, 18]. These papers also revealed that GPR84 activated chemotaxis and pro-inflammatory cytokine production in leukocyte and macrophage cell lines. Because some fatty acids regulate morphology, motility, and inflammatory responses of microglia [19–21], it is possible that a GPR84-mediated signal modulates microglial morphology and/or activity. Here, we addressed GPR84 function in primary cultured microglia using an endogenous ligand, capric acid (C10:0), a natural ligand, embelin (2,5-dihydroxy-3-undecyl-2,5-cyclohexadiene-1,4-dione), and a surrogate agonist, 6-n-octylaminouracil (6-OAU).

## Methods

### Animals

C57BL/6 and DBA/2 mice were purchased from SLC Japan (Hamamatsu, Japan). Mice were housed with food and water available ad-libitum in a temperature (23 ± 1 °C)- and humidity (50%)-controlled environment on a 12/12-h light/dark cycle (lights on at 09.00 h). All mice were maintained in accordance with the Guide for the Care and Use of Laboratory Animals (National Institutes of Health, 1996). The protocol for the animal experiment was approved by the Animal Ethics Committee of Nagoya University (Approval nos.: 26180, 27203, 28275).

### Materials

6-OAU, embelin, and capric acid were provided by Ono Pharmaceutical Co. (Osaka, Japan). 6-OAU and capric acid were dissolved in absolute ethyl alcohol, and embelin in dimethyl sulfoxide. Solutions were aliquoted to avoid freeze-thawing and stored at −80 °C. A selective Gi-coupled signaling inhibitor, pertussis toxin (PTX), was purchased from Wako (#168-22471; Osaka, Japan). Lipopolysaccharide (LPS) was purchased from Sigma-Aldrich (#L4516; St Louis, MO, USA).

### Microglial culture

Microglia were obtained from primary mixed cultures, in accordance with a previous report [22]. Briefly, meninges were removed from cerebra of C57BL/6 and DBA/2 mice at postnatal days 1–3. Cerebral tissue was dispersed with 0.25% trypsin (Invitrogen, Carlsbad, CA, USA) and DNase I (Roche Applied Science, Indianapolis, IN, USA). Cells were filtered through 100 μm nylon mesh, suspended in Dulbecco’s Modified Eagle’s medium (DMEM) supplemented with 10% fetal bovine serum (FBS) and 1% penicillin/streptomycin, and seeded into culture flasks. After 10–12 days in culture, detached microglia were collected and plated on 24- or 96-well culture dishes. Purity was > 98% [23].

### Quantitative real-time RT-PCR (qPCR)

Microglia were seeded into 96-well plates at a density of 3 × 10^4^ cells/well in DMEM containing 10% FBS and incubated overnight. After serum-starvation for 4 h, microglia were stimulated with 100 ng/mL LPS in the presence or absence of various 6-OAU concentrations for 16 h. SYBR Green Cells-to-Ct Kit (Thermo Fisher Scientific, Waltham, MA, USA) was used to synthesize cDNA in accordance with the manufacturer’s instructions. qPCR was performed using SYBR Green (Thermo Fisher Scientific) with the StepOnePlus Real-Time RT-PCR System (Thermo Fisher Scientific). The following primers were used: interleukin (IL)-1β forward: 5′-ACAGAATATCAACCAACAAGTGATATTCTC-3′; IL-1β reverse: 5′-GATTCTTTCCTTTGAGGCCCA-3′; IL-6 forward: 5′-ATCCAGTTGCCTTCTTGGGACTGA-3′; IL-6 reverse: 5′-TAAGCCTCCGACTTGTGAAGTGGT-3′; IL-12 p35 forward: 5′-GACATCACACGGGACCAA-3′; IL-12 p35 reverse: 5′-AGTCCTCATAGATGCTACCAAG-3′; IL-12 p40 forward: 5′-TTCACGTGCTCATGGCT-3′; IL-12 p40 reverse: 5′-TTGGTCCAGTGTGACCTT-3′; chemokine (C-X-C motif) ligand 1 (CXCL1) forward: 5′-ACCCAAACCGAAGTCATAGC-3′; CXCL1 reverse: 5′-TGGGGACACCTTTTAGCATC-3′; tumor necrosis factor (TNF)-α forward: 5′-AGCCGATGGGTTGTACCTTGTCTA-3′; TNF-α reverse: 5′-TGAGATAGCAAATCGGCTGACGGT-3′; inducible nitric oxide synthase (iNOS) forward: 5′-GGCAGCCTGTGAGACCTTTG-3′; iNOS reverse: 5′-GAAGCGTTTCGGGATCTGAA-3′; glyceraldehyde-3-phosphate dehydrogenase (GAPDH) forward: 5′-CAAGGTCATCCCAGAGCTGA-3′; and GAPDH reverse: 5′-CGGCACGTCAGATCCACGAC-3′. qPCR reaction conditions were: 1 cycle of 95 °C for 20 s, followed by 40 cycles of 95 °C for 3 s and 60 °C for 30 s. At the end of PCR, samples were subjected to melting analysis to confirm amplicon specificity. Relative gene expression (ΔCt value) was calculated based on threshold cycle (Ct) of the reference gene (GAPDH) and target gene (ΔCt = Ct_target gene_ − Ct_reference gene_). ΔΔCt was calculated as ΔCt_LPS_ _treatment_ − ΔCt_LPS + 6-OAU treatment_ with 2^-ΔΔCt^ determined as fold change [24].

### Staining cultured microglia

Microglia were seeded onto glass coverslips in 24-well culture dishes and incubated overnight at 37 °C. Cultured microglia were serum-starved for 4 h, and then stimulated with 6-OAU, embelin, and capric acid at the indicated concentration. For inhibition of Gi-coupled signaling, microglia were pretreated with PTX (100 ng/mL, 4 h). Microglia were fixed in phosphate-buffered saline (PBS), pH 7.4, containing 4% paraformaldehyde for 10 min at room temperature. Immunostaining was performed according to our previous report [23], using rabbit anti-nuclear factor kappa B (NF-kB) p65 antibody (1:100; #L1207; Santa Cruz Biotech, Santa Cruz, California, USA) and Alexa Fluor 488-labeled anti-rabbit IgG (1:1000; Invitrogen) as primary and secondary antibodies, respectively. Actin was visualized using Alexa Fluor 594-labeled phalloidin (1:100; #A12381; Invitrogen). Nuclei were stained with 4′,6-diamidino-2-phenylindole (DAPI) (1:5000; #340-07971; Dojindo Laboratories, Kumamoto, Japan). Membrane ruffling was visualized by staining of polymerized actin with phalloidin. Images were acquired by fluorescent microscopy (BZ-9000; Keyence, Osaka, Japan). Percentage of microglial ruffling was analyzed from 150 cells over three independent experiments. Line scan analysis was performed using ImageJ software (NIH, Bethesda, MD, USA).

### Time-lapse imaging and migration analysis of cultured microglia

Time-lapse imaging was performed as previously described [23]. Microglia were directly plated onto glass based dishes (#3971-035; Iwaki, Tokyo, Japan). Images were collected at 2-min intervals using an imaging microscope system (LCV110; Olympus, Tokyo, Japan). Cells were maintained at 37 °C in 5% CO_2_ throughout the experiment. Time-lapse images were used for quantitative analysis of morphology, as well as migration velocity and distance. Morphological changes were analyzed from 30 cells pooled from three individual experiments. Speed and distance of microglial migration were determined by tracking microglial cell bodies using MetaMorph 7.5.6.0 software (Universal Imaging, Media, PA, USA).

### Measurement of intracellular cAMP accumulation

Microglial cells were seeded at 3 × 10^4^ cells/well in 96-well plates and serum-starved for 4 h. Microglia were stimulated with 6-OAU for 30 min, subsequently treated with 50 μM forskolin (Calbiochem, La Jolla, CA, USA) for 20 min, and then extracted with 0.1 M hydrochloric acid. Extracts were centrifuged at 1000×*g* for 10 min, and supernatants were analyzed using the cAMP EIA kit (Cayman Chemical, Ann Arbor, MI, USA).

### Mutation verification

mRNA was isolated from DBA/2 mice, and cDNA obtained by reverse transcription. One primer pair was used—forward: 5′-GCTCAGATGCCAACTTCTCC-3′ and reverse: 5′-GACAACTGGCACCAAGACAA-3′. PCR was performed according to the manufacturer’s instructions (LA Taq; #RR002; Takara Bio, Otsu, Japan). PCR products were subjected to gel electrophoresis and bands collected for purification using the QIAquick gel extraction kit (#28704; Qiagen, Valencia, CA, USA). Extracted DNA was sequenced using the Big Dye Terminator Cycle Sequencing Ready Reaction Kit (Applied Biosystems, Foster City, CA, USA). Sequencing was performed using an ABI PRISM 310 Genetic Analyzer (Applied Biosystems) and the primer, 5′-AACAGCTCAGATGCCAACTTCTCCTGC-3′.

### Statistical analysis

All data are shown as mean ± standard error of the mean (SEM.). Statistical analyses were performed by one-way analysis of variance (ANOVA) with least significant difference (LSD), Dunnett’s (E) or T3 (3) tests using SPSS 20. For single comparisons between two groups, unpaired Student’s *t* test was used. Differences were considered significant for values of *p* < 0.05.

## Results

### GPR84 does not alter pro-inflammatory responses of cultured microglia

**Fig. 1:**
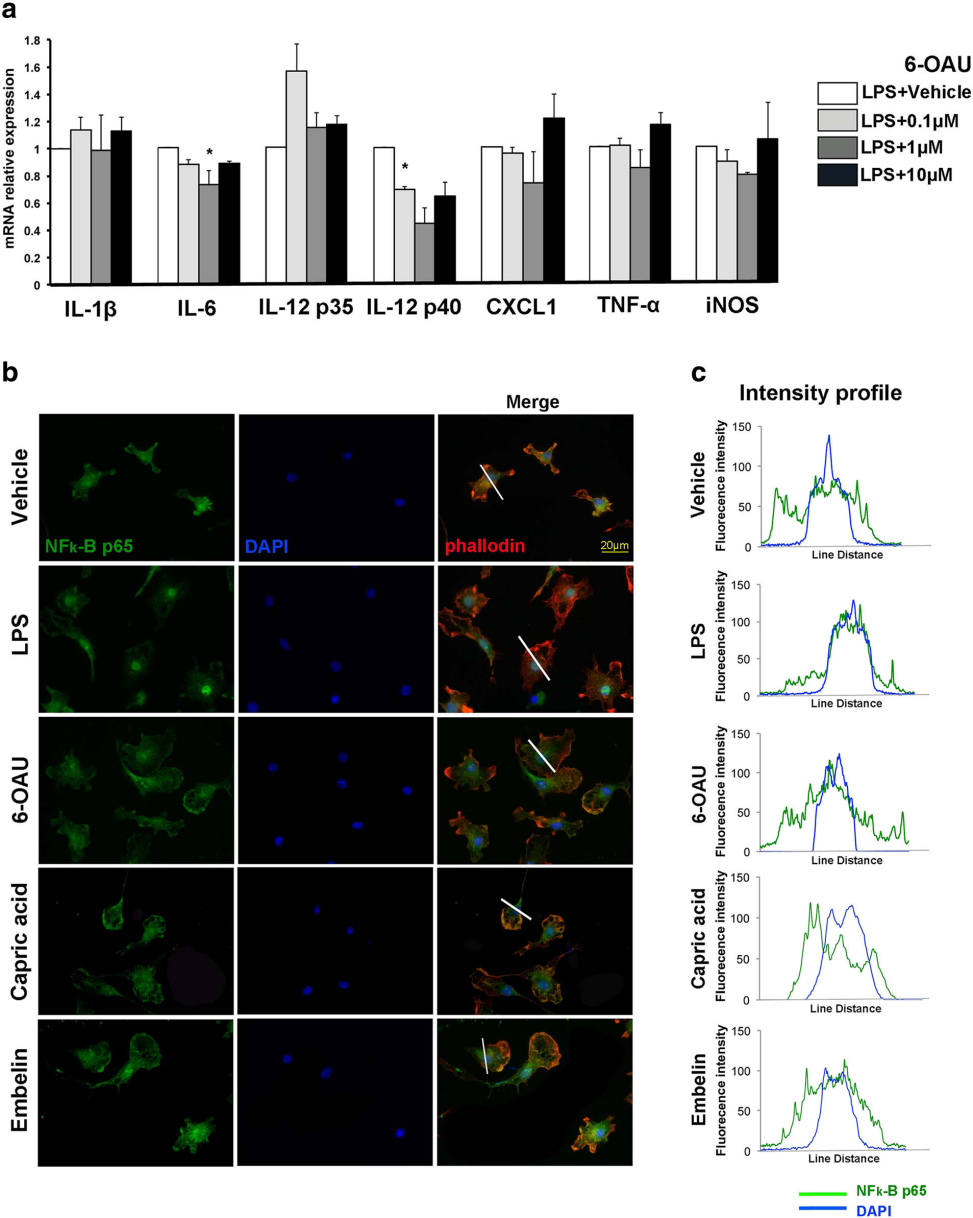
GPR84 does not modulate pro-inflammatory responses in cultured microglia. **a** Primary cultured microglia were treated with bacterial lipopolysaccharide (LPS) and either vehicle or 6-OAU for 16 h. mRNA for interleukin (IL)-1β, IL-6, IL-12 p35, IL-12 p40, chemokine (C-X-C motif) ligand 1 (CXCL1), tumor necrosis factor (TNF)-α, and inducible nitric oxide synthase (iNOS) was quantified by quantitative real-time RT-PCR. Results were normalized to LPS-treated samples. Values represent mean ± SEM from three independent experiments. *p < 0.05. **b** Microglia were treated with vehicle, 100 ng/mL LPS, 1 μM 6-OAU, 1 μM capric acid, and 1 μM embelin for 16 h. Cells were stained with anti-nuclear factor kappa B (NF-κB) p65 antibody (green), Alexa Fluor 594-labeled phalloidin (red), and DAPI (blue). **c** Line scan graphs of representative cells show the fluorescent intensity of p65 (green) and DAPI (blue) along white lines. Scale bar, 20 μm

Previous studies have reported a pro-inflammatory role for GPR84 in macrophages [17, 18]. Accordingly, we examined mRNA expression levels of pro-inflammatory cytokines including IL-1β, IL-6, IL-12 subunit p35 and p40, CXCL1 (a murine homolog of IL-8), TNF-α, and iNOS in LPS-treated primary cultured microglia after GPR84 agonist stimulation. Among surrogate agonists for GPR84, 6-OAU was used because of its potent activity [18]. Of all pro-inflammatory cytokines examined, mRNA expression was not up-regulated by 6-OAU treatment compared with vehicle. Rather, 6-OAU significantly suppressed mRNA expression of IL-6 (1 μM, *p* = 0.019) and IL-12 p40 (0.1 μM, *p* = 0.02) (Fig. 1a). Next, we examined additional inflammation-associated markers, specifically NF-κB. Nuclear translocation of NF-κB plays a critical role in pro-inflammatory gene expression [25, 26], therefore we examined subcellular localization of the NF-κB RelA subunit (p65) after 6-OAU treatment. Under normal culture conditions, p65 was spread throughout both the cytoplasm and nucleus of microglia, while LPS treatment prominently induced p65 translocation into the nucleus. In agreement with mRNA expression, 6-OAU as well as other GPR84 ligands, namely capric acid and embelin, did not cause nuclear translocation of p65 in microglia (Fig. 1b). Line scanning analysis determined the fluorescence intensity of intracellular p65 localization. p65 remained in the cytoplasm even after stimulation with GPR84, in contrast to nuclear localization in LPS-treated microglia (Fig. 1c). Taken together, GPR84-mediated signaling does not induce a pro-inflammatory phenotype in microglia in vitro.

### GPR84 agonists rapidly induce microglial ruffling

**Fig. 2:**
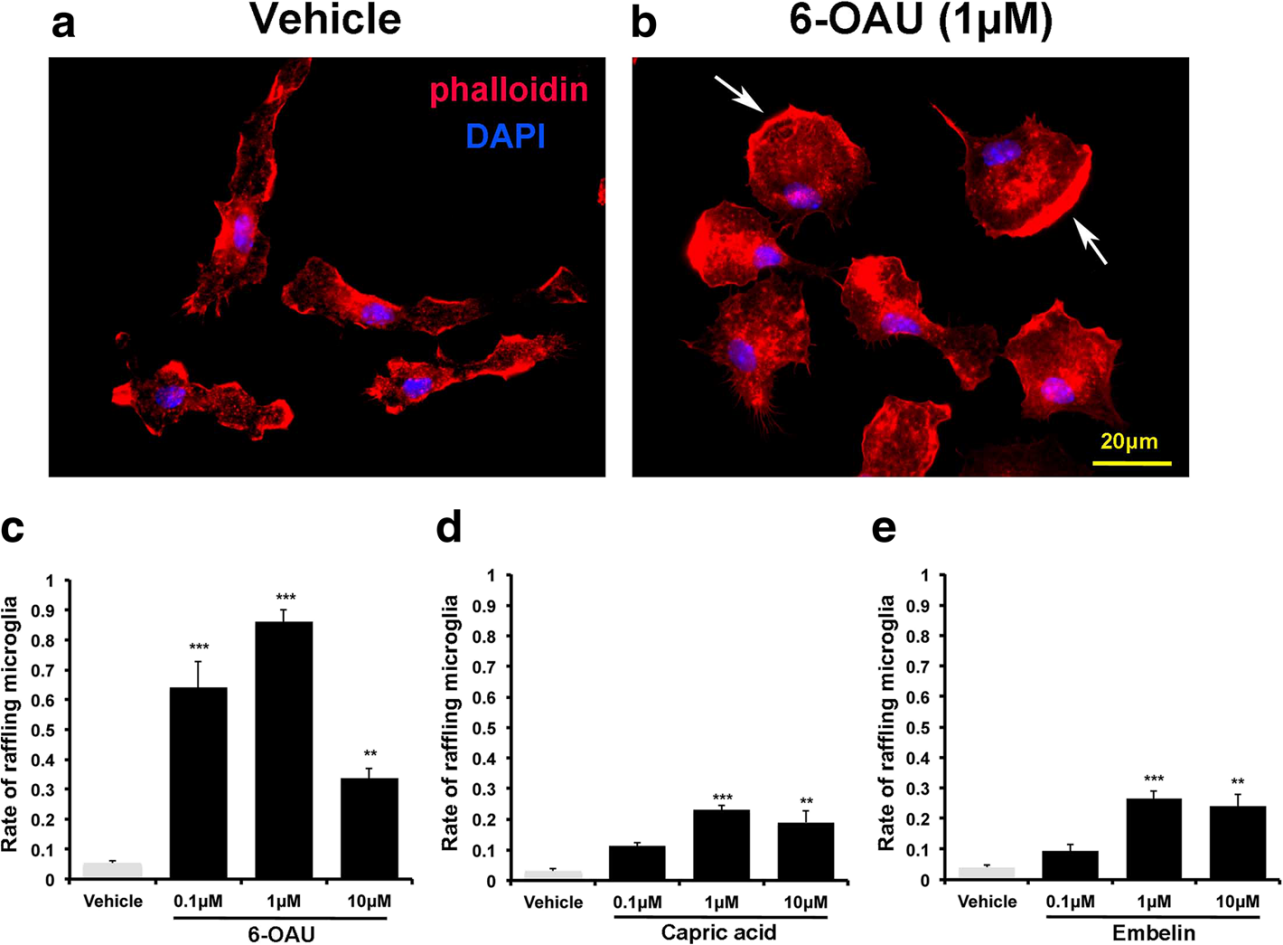
6-OAU, capric acid, and embelin induce microglial ruffling. **a**, **b** Microglia were incubated with vehicle and 6-OAU. After 2-min incubation, cells were fixed and stained with phalloidin (red) and DAPI (blue) to detect actin polymerization (white arrows) and nuclei, respectively. Scale bar, 20 μm. **c**–**e** Rate of microglia undergoing membrane ruffling 2 min after stimulation with vehicle, 6-OAU, capric acid, and embelin. The percentage of microglial rufflings was calculated by staining actin-polymerized lamellipodia with phalloidin [27]. Data represent mean ± SEM. In total, 150 cells from at least three independent experiments were used for quantification. **p < 0.01, ***p < 0.001

Next, we examined morphological changes in microglia (Fig. 2). 6-OAU induced apparent lamellipodia formation in cultured microglia (Fig. 2a, b). Microglia exhibited rounded cell bodies, with the cytoplasmic membrane showing sheet-like membrane protrusions that were heavily stained with phalloidin. Microglial ruffling was also observed using the endogenous ligand, capric acid, and a natural ligand, embelin. The percentage of microglial rufflings was calculated by staining actin-polymerized lamellipodia with phalloidin [27]. Two minutes after agonist application, the highest percentage of ruffling was observed with 6-OAU (6-OAU at 1 μM, 86%; capric acid at 1 μM, 23%; embelin at 1 μM, 26.4%) (Fig. 2c–e). Efficiency of the three agonists on microglial ruffling correlates with cAMP suppression activity shown in a previous study [18]. Thus, we primarily used 6-OAU for further research.

### Microglial motility is induced by GPR84 agonists

**Fig. 3:**
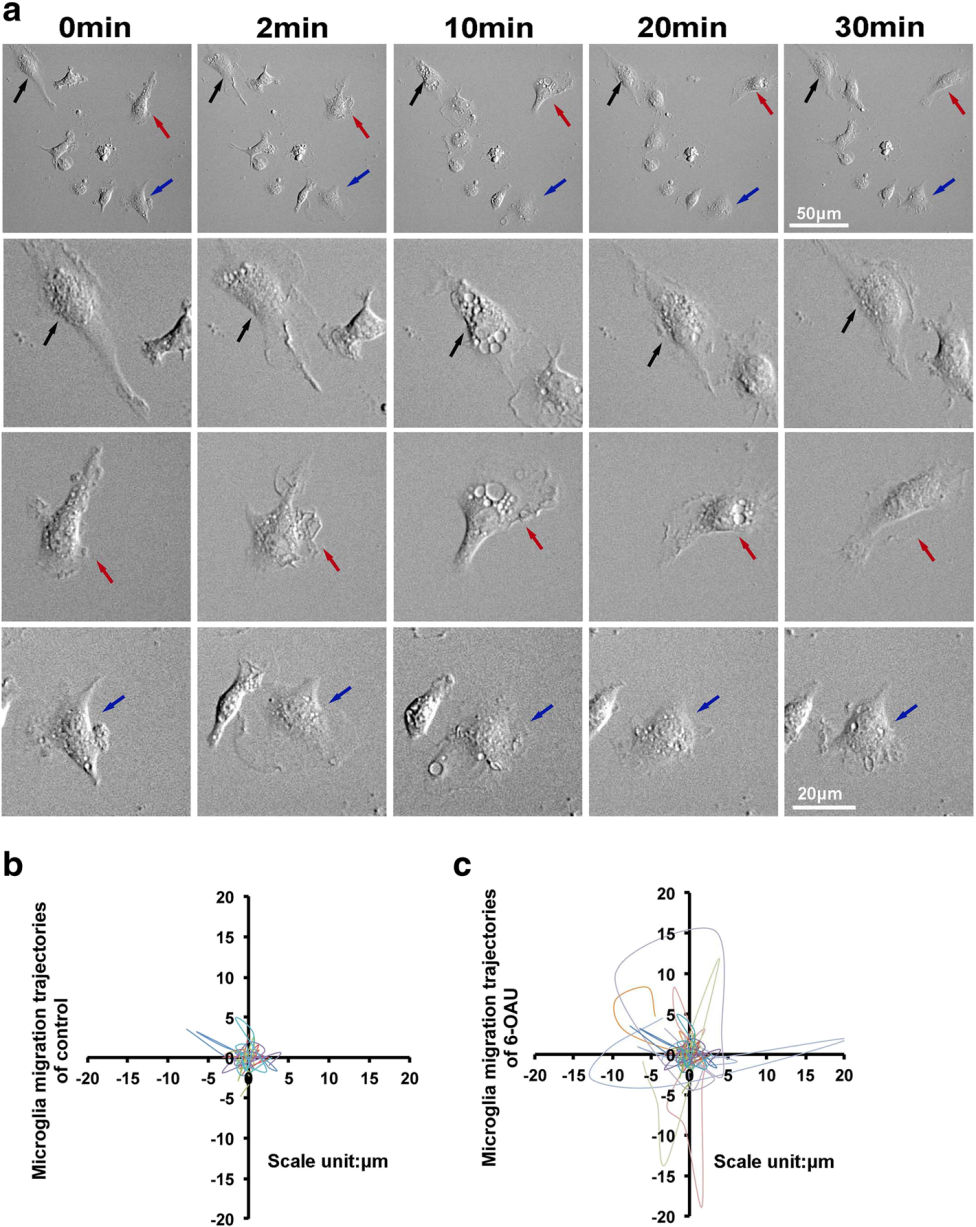
Time-lapse analysis of 6-OAU-treated microglia. **a** Microglia were exposed to 1 μM 6-OAU, and live imaging was performed for 30 min. The second, third, and fourth rows show higher magnifications of cells indicated by arrows in the top row. Scale bar, 50 μm (top row) and 20 μm (other rows). **b**, **c** Overlay of individual microglial trajectories after control or 6-OAU treatment for 30 min (*n* = 5 each). The starting point of each cell was aligned to the same position (*x* = 0, *y* = 0)

**Fig. 4:**
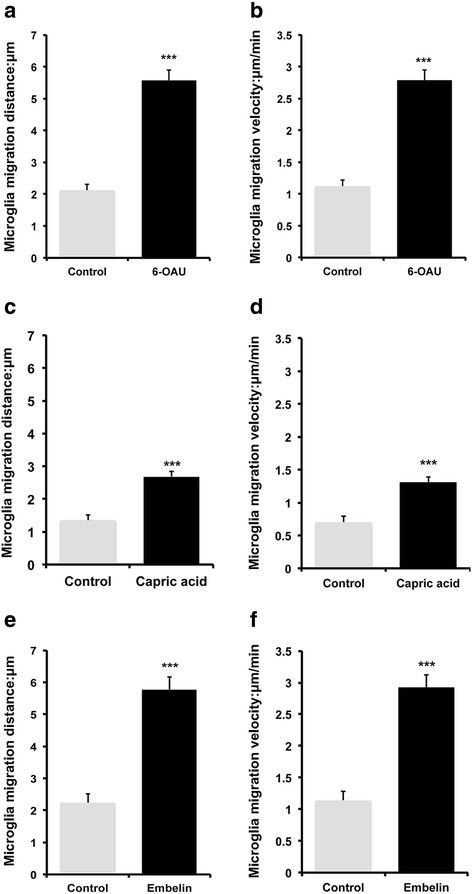
Quantification of cell migration distance and velocity. Cells were tracked over 30-min periods and MetaMorph-based numerical values calculated. Microglia were exposed to 1 μM 6-OAU (**a**, **b**), capric acid (**c**, **d**), and embelin (**e**, **f**), with migration distance (**a**, **c**, **e**) and velocity (**b**, **d**, **f**) calculated. Data represent mean ± SEM of 15 (**a**, **b**), 13 (**c**, **d**), and 30 (**e**, **f**) cells per group. ***p < 0.001

Membrane ruffling is associated with microglial motility [28], therefore we examined motility of 6-OAU-treated microglia using time-lapse imaging (Fig. 3). 6-OAU induced ruffling, as shown in Fig. 2. 6-OAU treatment also increased microglial motility over a relatively short period, which was demonstrated by re-plotting microglial migration trajectories (Fig. 3b, c). Measurement of distance and velocity clearly indicated that 6-OAU induced higher motility (Fig. 4a, b). A similar response was observed in capric acid- (Fig. 4c, d) and embelin- (Fig. 4e, f) treated microglia, indicating that GPR84-mediated signals enhance microglial motility.

### 6-OAU-induced microglial motility is mediated by a PTX-sensitive Gi/o pathway

**Fig. 5:**
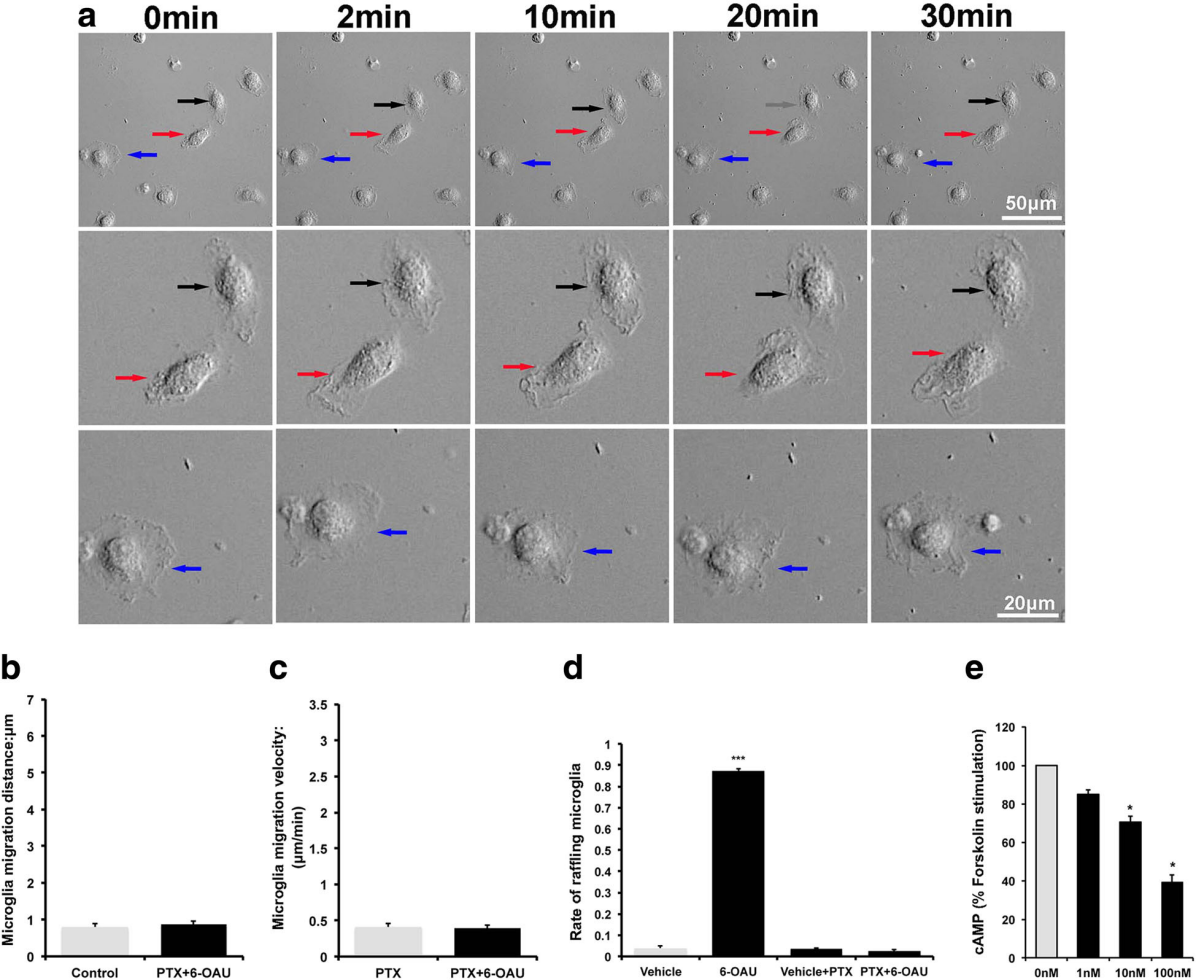
Effect of pertussis toxin (PTX) on 6-OAU treated microglial motility. **a** Microglia were pretreated with PTX (100 ng/mL) for 4 h and then exposed to 1 μM 6-OAU. Live imaging was performed for 30 min. The second and third rows show higher magnifications of cells indicated by arrows in the top row. Scale bar, 50 μm (top row) and 20 μm (other rows). **b**, **c** MetaMorph-based quantification of distance and velocity of cell migration. Cells were tracked over 30-min periods. Data represent mean ± SEM of 15 cells per group. **d** Rate of microglia undergoing membrane ruffling in each condition. Microglia were incubated with vehicle or 1 μM 6-OAU for 2 min in the presence or absence of PTX (100 ng/mL). Data represent mean ± SEM. In total, 150 cells from at least three independent experiments were used for quantification. **e** Microglia were cultured in 96-well plates and treated with 50 μM forskolin for 20 min after 6-OAU treatment. cAMP levels were determined using the cAMP EIA kit and presented as mean ± SEM of three independent experiments. *p < 0.05, ***p < 0.001

GPR84 activation by its ligands is coupled to a PTX-sensitive Gi/o pathway [18]. Hence, we examined the involvement of the Gi/o pathway. PTX completely suppressed 6-OAU-induced microglial motility, such as distance and velocity (Fig. 5a–c). We also found that membrane ruffling of cultured microglia exposed to 6-OAU was completely blocked (Fig. 5d). In support of Gi/o-coupling of GPR84, we confirmed that 6-OAU dose-dependently inhibited forskolin-stimulated cAMP production in cultured microglia (Fig. 5e). Altogether, these results suggest that GPR84 activation by 6-OAU couples primarily to a PTX-sensitive Gi/o pathway.

### GPR84-deficient microglia fail to respond to 6-OAU

**Fig. 6:**
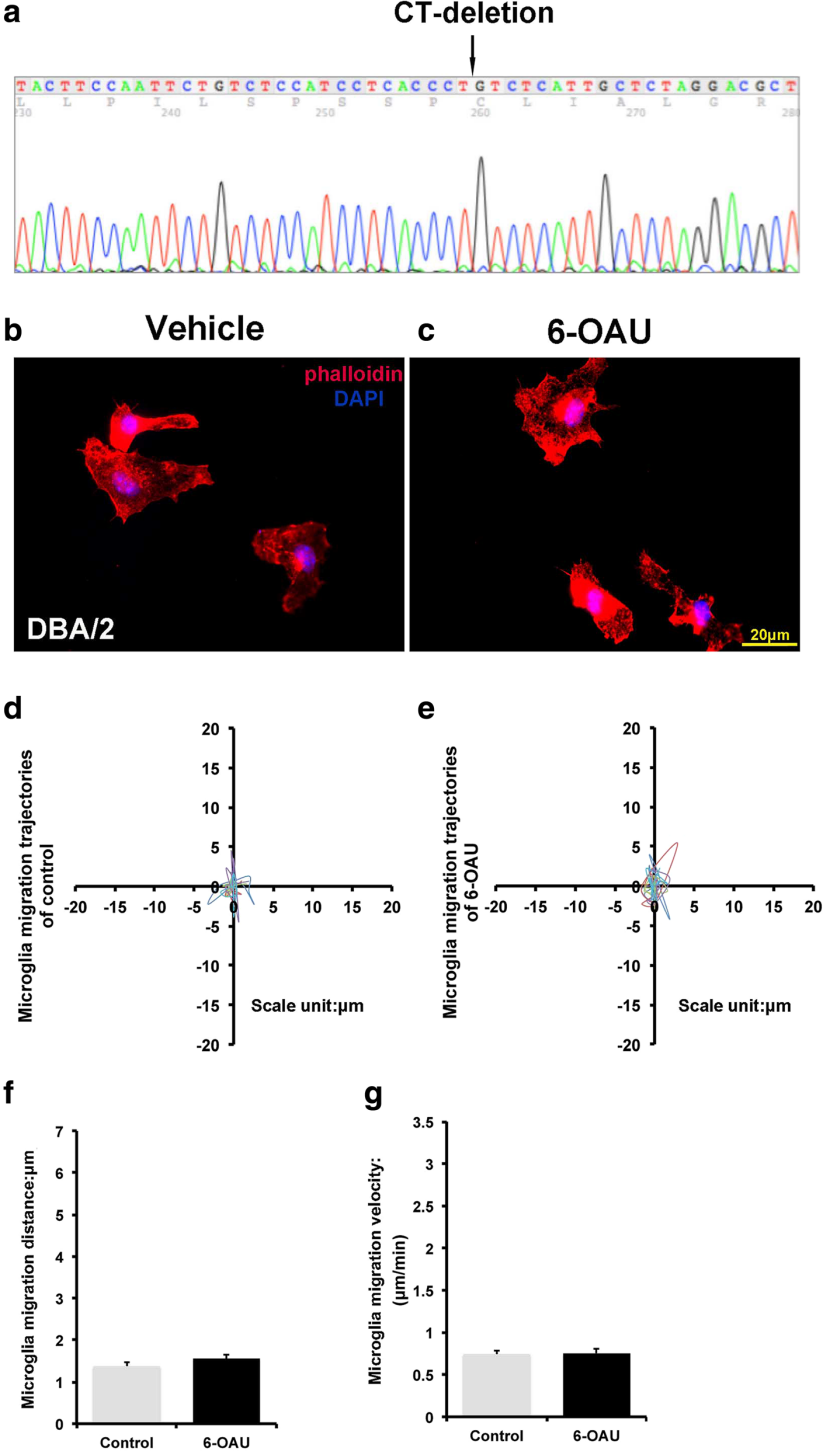
Motility analysis of 6-OAU-treated microglia from DBA/2 mice. **a** DNA sequencing shows a frame-shift deletion in exon 2 of *GPR84* from DBA/2 mice. **b**, **c** Microglia from DBA/2 mice were exposed to vehicle and 1 μM 6-OAU. After 2-min incubation, cells were fixed and stained with phalloidin (red) and DAPI (blue) to detect actin polymerization and nuclei, respectively. Scale bar, 20 μm. **d**, **e** Overlay of individual microglial trajectories after vehicle or 6-OAU treatment for 30 min (*n* = 5 each). **f**, **g** MetaMorph-based quantification of distance and velocity of cell migration. Cells were tracked over 30-min periods. Data represent mean ± SEM of 10 cells per group

To further confirm that motility changes induced by 6-OAU are GPR84 dependent, we used the DBA/2 mouse strain, which has a 2-bp frame-shift deletion in the second exon of the *GPR84* gene. The deletion results in a premature stop codon and predicted truncated protein [11]. We sequenced exon 2 of *GPR84* using DBA/2 mice cDNA to confirm the 2-bp frame-shift deletion (Fig. 6a). 6-OAU-induced membrane ruffling did not occur in microglia from DBA/2 mice (Fig. 6b, c). As expected, 6-OAU-induced motility was also blocked, with migration distance and velocity unchanged after 6-OAU treatment in DBA/2 microglia (Fig. 6d–g). These findings indicate that GPR84 is involved in microglial ruffling and motility induced by 6-OAU.

## Discussion

In the present study, we show that GPR84 mediated signaling induces microglial ruffling and motility via the Gi/o pathway (Figs. 2, 3, 4, 5, and 6), but does not alter pro-inflammatory responses in vitro. These responses may be characteristic of GPR84 signaling in microglia, and distinct from macrophages.

GPR84 is a seven-transmembrane GPCR, initially identified as an orphan receptor [9]. Later, the [^35^S] GTPγS binding assay, which can detect Gαi pathways, demonstrated that medium-chain fatty acids, especially capric acid (a 10 carbon chain fatty acid), have the most potent activity for GPR84 in vitro [17]. Expression of GPR84 is mainly seen in myeloid cells (e.g., monocytes, macrophages, microglia, and neutrophils) and is up-regulated by immune stimuli [7, 29, 30]. In the central nervous system (CNS), several transcriptome studies have reported GPR84 as one of the signature microglial genes [31, 32]. In addition, peripheral nerve injury induced GPR84 expression in microglia [3, 13], leading us to propose that GPR84 is important for microglial regulation, although its precise function remains to be determined.

Potent surrogate agonists are a useful tool for characterizing functionally unknown or less known GPCRs. Suzuki et al. [18] screened an in-house chemical library with the [^35^S]GTPγS binding assay and identified a highly effective agonist compound, 6-OAU, which activated human GPR84 in the presence of Gqi5 chimeric G proteins. Furthermore, EGFP-labeled human GPR84 internalization was observed in a 6-OAU-dependent manner. Altogether, these findings suggest that 6-OAU activates GPR84 [18]. GPR84 is proposed to be a pro-inflammatory receptor that induces pro-inflammatory cytokine expression [13, 17, 18]. GPR84 is expressed selectively in immune cells [7, 17], and Suzuki et al. have shown that GPR84 activation amplifies LPS-stimulated IL-8 production in human polymorphonuclear leukocytes and TNF-α production in macrophages [18]. Concomitantly in a peripheral nerve injury-induced neuropathic pain model, GPR84 deficiency induced arginase 1 expression in macrophages that infiltrated peripheral nerve and reduced neuropathic pain behavior [13]. Collectively these experiments suggest that GPR84 mediates pro-inflammatory signaling, at least in macrophages. However, in microglia, mRNA expression of pro-inflammatory cytokines was not induced by the agonists, 6-OAU, capric acid, and embelin (Fig. 1a). In addition, these agonists did not cause nuclear translocation of NF-κB, which elicits inflammatory microglial responses (Fig. 1b) [26, 33], suggesting that GPR84 is not a pro-inflammatory receptor in microglia. Instead, here we demonstrate GPR84 as an activator of motility in microglia (Figs. 2, 3, 4, 5, and 6). Because Suzuki et al. showed that 6-OAU stimulates chemotaxis of macrophages both in vitro and in vivo [18], induction of motility by GPR84 is shared between macrophages and microglia. GPR84 uses a PTX-sensitive Gi/o downstream signaling pathway to induce motility of both macrophages [18] and microglia [8] (Fig. 5). Although downstream receptor signaling appears to be the same between microglia and macrophages, microglial inflammatory responses failed to respond to agonist stimuli (Fig. 1). We cannot explain this, but additional unknown signaling pathways may exist downstream of the receptor.

Several fatty acids regulate microglial morphology and activity [19–21]. Given that medium-chain fatty acids are endogenous ligands for GPR84 [17, 18], fatty acids released under inflammatory, traumatic, and degenerative events within the CNS [34–36] may trigger activation of microglial GPR84. In this context, microglial GPR84 appears to be involved in activation of microglial motility in response to CNS insults [8]. This is suggested by the fact that peripheral nerve injury promotes GPR84 expression in microglia and induces microglial migration towards injured neurons [3].

This study demonstrates the functional significance of GPR84 agonists in microglia in vitro. Our findings show that GPR84 agonist-mediated signaling affects morphology and motility of microglia, suggesting that microglial GPR84 could be a therapeutic target in microglia-associated diseases such as multiple sclerosis and Alzheimer’s disease [8]. Further development of both agonists and antagonists will provide increased understanding of GPR84 function as well as therapeutic tools for neuronal inflammatory, traumatic, and degenerative diseases.

## Conclusions

The present study demonstrates that GPR84 does not modulate pro-inflammatory responses in primary cultured microglia. However, endogenous and natural ligands and a surrogate agonist (capric acid, embelin, and 6-OAU, respectively) for GPR84 rapidly induce microglial ruffling and motility. 6-OAU induced activation of microglia was suppressed by PTX, which inhibits the Gi/o pathway located downstream of GPR84. Concomitantly, 6-OAU failed to induce membrane ruffling and motility in primary cultured microglia from DBA/2 mice lacking functional GPR84. Since GPR84 is known as a receptor for medium-chain fatty acids such as capric acid, fatty acids released from damaged brain cells may act on microglial GPR84 to increase cellular motility.
